# Trabecular Bone Score as a Complementary Tool for the Assessment of Bone Mineral Density in Patients with Asymptomatic Monoclonal Gammopathies

**DOI:** 10.3390/jcm13216461

**Published:** 2024-10-28

**Authors:** Styliani Drakoulidou, Ioannis Ntanasis-Stathopoulos, Aikaterini Kyritsi, Vassilis Koutoulidis, Panagiotis Malandrakis, Nikolaos Kanellias, Efstathios Kastritis, Meletios A. Dimopoulos, Maria Gavriatopoulou, Athanasios Chalazonitis, Evangelos Terpos

**Affiliations:** 1 Department of Radiology, Alexandra Hospital, 11528 Athens, Greece; 2 Department of Clinical Therapeutics, School of Medicine, National and Kapodistrian University of Athens, 11528 Athens, Greece; 3 Department of Pharmacy, School of Health Sciences, National and Kapodistrian University of Athens, 11527 Athens, Greece; 4 First Department of Radiology, School of Medicine, Areteion Hospital, National and Kapodistrian University of Athens, 11528 Athens, Greece; 5 Department of Medicine, Korea University, Seoul 02841, Republic of Korea

**Keywords:** trabecular bone score, bone mineral density, fracture, DXA scan, monoclonal gammopathy of undetermined significance, smoldering myeloma

## Abstract

**Background/Objectives:** Monoclonal gammopathies, such as Monoclonal Gammopathy of Undetermined Significance (MGUS) and Smoldering Multiple Myeloma (SMM), are conditions marked by the overproduction of specific monoclonal proteins. Patients with these conditions are known to have a higher risk of fractures compared to the general population, yet there are no established guidelines for assessing or managing their skeletal health. The Trabecular Bone Score (TBS), which can be calculated from DXA images of the lumbar spine, provides additional insights into bone microarchitecture. **Methods:** This study aimed to determine whether TBS can serve as a supplementary tool in assessing bone loss in MGUS and SMM patients. Conducted from 2020 to 2023, the study involved 148 participants—74 diagnosed with a myeloma precursor state and 74 healthy controls—who underwent simultaneous DXA and TBS measurements. **Results:** The results indicated a weak positive correlation (R = 0.405) between DXA and TBS T-scores, suggesting that other factors may influence the measurements. When analyzed separately, the correlations remained weak for both MGUS (R = 0.250) and SMM (R = 0.485). Interestingly, discrepancies were noted in T-score classifications; for instance, a patient classified as normal via DXA could be deemed osteopenic or osteoporotic with TBS. **Conclusions:** Overall, the findings suggest that incorporating TBS alongside DXA can enhance the accuracy of bone density assessments, facilitating earlier diagnosis and treatment initiation for osteoporosis in asymptomatic patients with monoclonal gammopathies.

## 1. Introduction

Monoclonal gammopathies are disorders involving a clone of abnormal plasma cells that may secrete a particular immunoglobulin or its components. The presence, level, and type of immunoglobulin play a role in the diagnosis, staging, and treatment of the disease. Smoldering Multiple Myeloma (SMM) is an asymptomatic condition that lies between Monoclonal Gammopathy of Undetermined Significance (MGUS) and Multiple Myeloma (MM) along the spectrum of clonal plasma cell proliferative disorders [1,2,3,4,5].

SMM progresses to MM at a rate of about 10% per year for the first five years, 3% per year for the next five years, and 1.5% per year thereafter [6,7]. They present with monoclonal protein ≥ 3 g/dL, clonal plasma cells in the bone marrow ≥ 10% and a higher disease burden than MGUS, but without symptoms of organ damage or myeloma-defining events. MGUS is characterized by an M protein concentration in the serum of <3.0 g/dL, <10% clonal plasma cells in the bone marrow, and the absence of end-organ damage (hypercalcemia, renal insufficiency, anemia, and bone lesions) or myeloma-defining events. MGUS occurs in approximately 1–2% of adults, with a prevalence of over 3.2% in the general Caucasian population over 50 years of age. Men appear to have a higher risk of developing MGUS compared to women, with rates of 3.7% versus 2.9%, respectively [6]. There is now clear evidence from epidemiological studies indicating that individuals with MGUS have a higher risk of fractures [7,8,9,10,11,12,13,14,15,16], and the prevalence of MGUS is higher in patients with osteoporosis [13,17,18]. The underlying etiology for the increased risk for fractures may be associated with MGUS-related osteoporosis/bone disease, MGUS progression to symptomatic myeloma, and/or comorbidities including peripheral neuropathy that may create a predisposition to falls [8,19].

TBS (Trabecular Bone Score) is a texture parameter that can be derived from DXA images of the lumbar spine and quantifies local variations in pixel intensities. TBS is derived from experimental variograms obtained from the grayscale levels of a DXA image. It has been shown that TBS is related to the structural condition of bone microarchitecture [20,21,22,23,24].

TBS is calculated within a few seconds using the images obtained from the DXA scan for determining BMD, utilizing the TBS iNsight^®^ software version 3.0 installed on bone density measurement units. TBS is determined after the bone mineral density (BMD) measurement and at the same region of interest. In 2023, the European Society on Clinical and Economic Aspects of Osteoporosis, Osteoarthritis, and Musculoskeletal Diseases (ESCEO), the International Osteoporosis Foundation (IOF), and the Collaborating Center for Epidemiology of Musculoskeletal Health and Aging presented a review of the updated scientific literature, providing consensus, expert statements, and corresponding operational guidelines on the clinical use of TBS in the management of osteoporosis [20]. Overall, 96 articles containing data on the use of TBS in fracture risk prediction in men and women from more than 20 countries were reviewed. The updated results indicate that TBS enhances fracture risk prediction in both primary and secondary osteoporosis. When combined with BMD and clinical risk factors, TBS can provide additional information for initiating and selecting osteoporosis treatment [20,25].

Because TBS reflects the bone microarchitecture and specifically the bone quality, the present study attempts to assess whether the TBS values further assist in the stratification of the fracture risk in individuals with asymptomatic monoclonal gammopathies.

## 2. Materials and Methods

### 2.1. Subjects

The study focused on a specific population of patients diagnosed with asymptomatic monoclonal gammopathies, specifically monoclonal gammopathy of undetermined significance (MGUS) and smoldering multiple myeloma (SMM). The patient inclusion criteria were:

1. Diagnosis of Asymptomatic MGUS or SMM:

Patients must have a confirmed diagnosis of either MGUS or SMM according to the established International Myeloma Working Group criteria [26,27]. This includes the presence of monoclonal protein in the serum or urine, a low level of monoclonal plasma cells in the bone marrow, and no evidence of related organ or tissue impairment. This criterion ensures that only patients who are asymptomatic and meet specific laboratory thresholds are included, allowing for a clearer understanding of the natural history of these conditions.

2. Consecutive Selection:

The study included 74 consecutive patients diagnosed between 2020 and 2023. This approach helps minimize selection bias, ensuring that the sample reflects a typical cohort of patients seen in the reference center during this period.

3. Simultaneous Diagnostic Imaging:

All patients underwent dual-energy X-ray absorptiometry (DXA) scans of the lumbar spine and non-dominant hip to assess bone mineral density and TBS measurements simultaneously. This criterion ensures that the study could adequately evaluate the impact of asymptomatic monoclonal gammopathies on bone health.

4. Comprehensive Medical History:

A thorough medical history was necessary to identify any potential exclusions related to prior treatments or existing medical conditions, ensuring that the focus remained on the effects of MGUS and SMM without the confounding effects of other diseases or treatments.

For the control, the selection criteria were the same as above, except for the diagnosis of asymptomatic MGUS and SMM.

Patients and controls who did not meet all criteria or lacked information were excluded from the study. The exclusion criteria entailed:

1. Current or previous treatment with any bone-directed agent (such as bisphosphonates, denosumab, teriparatide, or abaloparatide).
2. Presence of endogenous or exogenous glucocorticoid excess (including glucocorticoid therapy at an equivalent dose of prednisone ≥7.5 mg/day for 3 months or more).
3. Presence of primary or metastatic bone neoplasm.
4. Presence of primary or secondary bone marrow neoplasm.
5. Symptomatic multiple myeloma or pathology related to monoclonal gammopathy requiring treatment.
6. Presence of chronic kidney disease stage 3 or greater, including end-stage renal disease.
7. Presence of rheumatoid arthritis.

### 2.2. Matching

The control group was randomly selected from people who needed a BMD measurement and did not have a diagnosis of MGUS and SMM. In addition, they met all of the aforementioned exclusion criteria.

Each patient and control case were matched 1:1 in terms of gender, height, weight, body mass index (BMI), smoking status, and dairy product consumption.

### 2.3. BMD

BMD measurements were performed using DXA with a Hologic Horizon Bone Densitometry System User Guide MAN-04871, Revision 005. To perform TBS analyses, all individuals were measured at the lumbar spine and the non-dominant hip. All scans were interpreted according to the guidelines of the International Society for Clinical Densitometry. The generated T-scores were stratified according to WHO criteria into normal (T-score ≥ −1.0), osteopenia (−1.0 > T-score > −2.5), or osteoporosis (T-score ≤ −2.5) [28].

### 2.4. TBS

TBS measurements were obtained on lumbar spine DXA images using the TBS iNsight software version 3.0 (Medimaps, Merignac, France). The TBS values were categorized as low (TBS ≤ 1.23), intermediate (1.23 < TBS ≤ 1.31), or normal (TBS > 1.31), in accordance with the current literature [20].

### 2.5. Statistical Analyses

Data was collected from the patient and the control groups. After the data collection, descriptive statistics were utilized. In this study, the variables were categorical (nominal, ordinal) or scale. A normality test was also conducted to determine whether the data had been drawn from a normally distributed population based on Kolmogorov–Smirnov criteria. Based on the normality test results, all variables except for height, hip total T-score, TBS, and TBS T-score were found to follow a non-parametric distribution (*p*-value < 0.05). Subsequently, statistical comparisons between groups were conducted. Specifically, either an independent sample *t*-test for parametric measurements or the Mann–Whitney U test for non-parametric measurements was applied to calculate differences between groups of participants. Overall, *p*-values < 0.05 were considered statistically significant and the statistical power was set at 0.80.

The correlation coefficient (R) was used to investigate the strength and direction of the relationship between TBS T-score values and T-score values of the total hip, hip neck, and L1–L4. In both groups (patient–control), either the parametric Pearson method or the non-parametric Spearman method was applied. A correlation of R = 0.9 indicates a strong positive association between two variables, whereas a correlation of R = −0.2 indicates a weak negative association.

Moreover, chi-square and multiple correspondence analysis (MCA) were performed to investigate the associations between patients’ characteristics. The chi-square analysis was used to investigate the relationships between two or more features (at the 5% nominal significance level). When more than two hypothesis tests were performed simultaneously, the Bonferroni correction was used. MCA is a machine learning technique used to analyze categorical data by representing associations among variables in a lower-dimensional space. In the MCA plot, the categorical variables are represented as points. The origin of the plot represents the average of all the data points. The vector line direction indicates the association between the overall average and each variable, while the vector line length represents the strength of that association. Also, if two vector lines are closer together, it suggests that the variables they represent are positively associated. Conversely, if they are far apart, it suggests a negative association. MCA plots can reveal interesting relationships among categorical variables [29].

In this study, all statistical analyses were performed with IBM SPSS v.28 (Chicago, IL, USA).

## 3. Results

### 3.1. Demographic Characteristics and Participant Features

A total of 148 participants were enrolled in the study. Of these, 74 (50.0%) were patients with asymptomatic monoclonal gammopathy and 74 (50.0%) were healthy controls.

Among patients, 46 (62.2%) were diagnosed with SMM and 28 (37.8%) with MGUS; whereas 27 (36.5%) were men and 47 (63.5%) were women. In the patient cohort, the median age was 65.5 years, the median height was 161 cm, the median weight was 74.5 kg, and the median BMI was 28.8 kg/m^2^. Among all patients, 42 (56.8%) were over 65 years old and 60 (81.1%) were obese (BMI ≥ 25). Most of the patients (n = 57 or 77.0%) were non-smokers, 8 (10.8%) had a family history of osteoporosis, and 27 (36.5%) mentioned regular consumption of dairy products.

Among the healthy controls, 27 (36.5%) were men and 47 (63.5%) were women. In this group, the median age was 62 years, the median height was 161.5 cm, the median weight was 72 kg, and the median BMI was 27.3 kg/m^2^. Only 26 (35.1%) of them were over 65 years old and 52 (70.3%) were obese (BMI ≥ 25). The majority (60 or 81.1%) were non-smokers, 6 (8.1%) had family history of osteoporosis, and 29 (39.2%) mentioned regular consumption of dairy products (Table 1). There were no statistically significant differences in the baseline characteristics between the two groups (patients–controls) (*p* > 0.05), except for age (*p* < 0.05).

### 3.2. Correlation Between DXA T-Score and TBS T-Score

**Figure 1:**
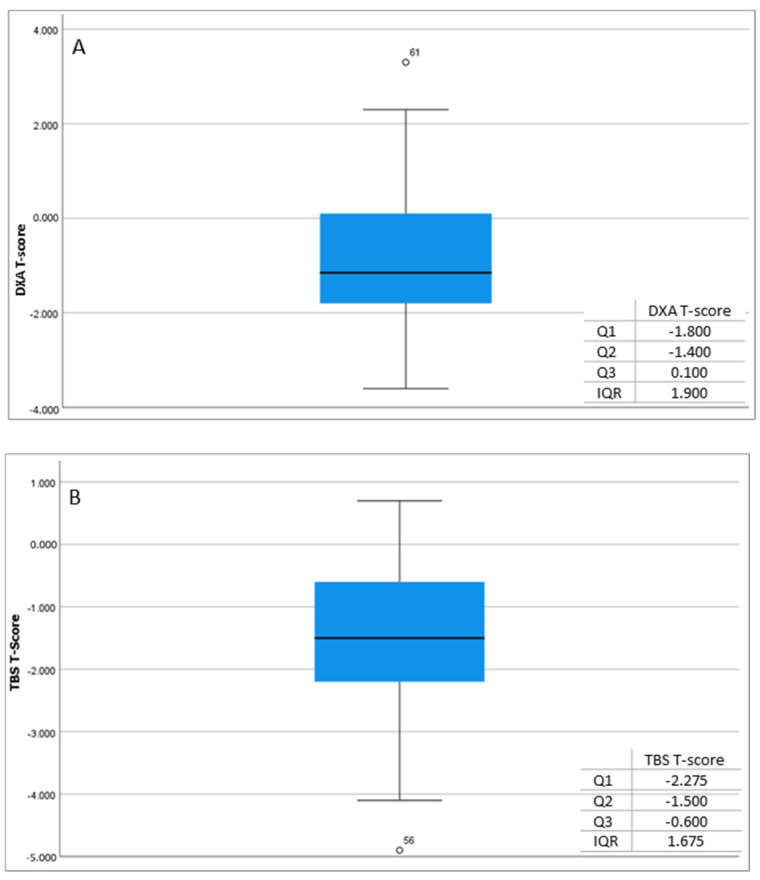
Measurements of DXA T-score (**A**) and TBS T-score (**B**) in patients.

In the patient cohort, the relationship between the two variables, i.e., the DXA T-score and TBS T-score, was described by the correlation coefficient R = 0.405, which indicates a weak positive correlation between the two variables. Based on the square of the correlation R^2^ = 0.164, the percentage variance of the entire dataset is only 16.4%, suggesting that there is a discrepancy between these two scores. This implies that the supplemental tool of TBS may lead to a distinct assessment of each patient’s BMD (Figure 1).

**Figure 2:**
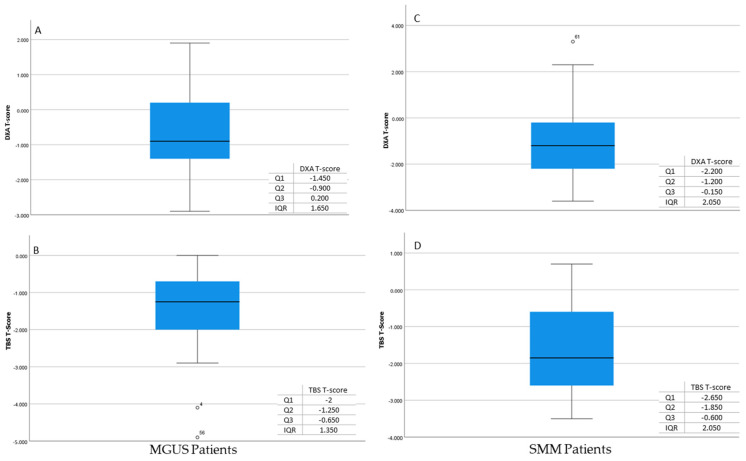
Measurements of DXA T-score (**A**) and TBS T-score (**B**) in MGUS patients. Measurements of DXA T-score (**C**) and TBS T-score (**D**) in SMM patients.

Similar results were observed when the patients were subdivided into the subgroups of MGUS and SMM. In the MGUS group, the correlation coefficient was found to be R = 0.250, which also indicates a weak positive correlation between the two variables. The percentage variance of this group was only 6.2%, which means the two variables differ from each other. Similarly, in the SMM group, the relationship between the two variables was found to be a weak positive correlation, with R = 0.485. Based on the square of the correlation, R^2^ = 0.235, the % variance of the data was only 23.5%, which means the two variables differ from each other (Figure 2).

**Figure 3:**
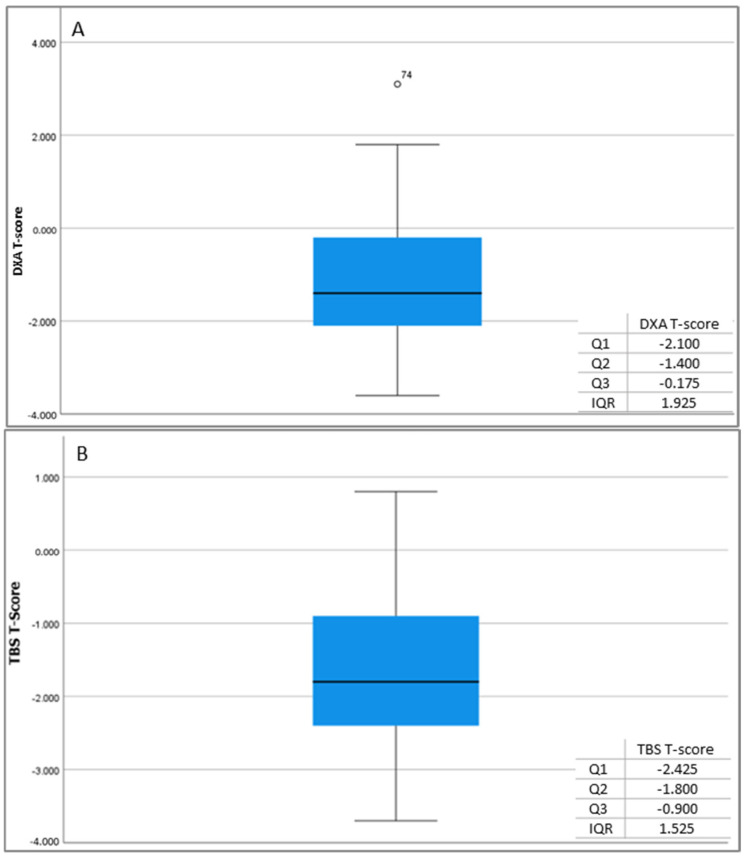
DXA T-score (**A**) and TBS T-score (**B**) measurements in controls.

Analogous results were observed in the control group, where the correlation coefficient (R = 0.324) and the squared correlation (R^2^ = 0.105) were also found to be very low. These findings also indicate a discrepancy between the DXA T-score and the TBS T-score (Figure 3).

**Figure 4:**
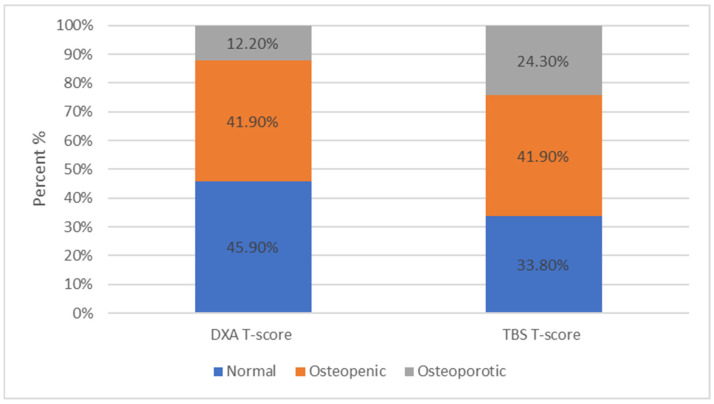
Among all patients, 34 (45.9%) were within normal limits, 31 (41.9%) were characterized as osteopenic, while only 9 (12.2%) were osteoporotic based on the DXA scan. Among all patients, 25 (33.8%) were within normal limits, 31 (41.9%) were characterized as osteopenic, and 18 (24.3%) as osteoporotic based on the TBS evaluation.

Furthermore, it was observed that the patients measured with DXA and categorized by the T-score into normal, osteopenic, and osteoporotic changed classification categories when TBS measurements were applied. Specifically, among all patients, 45.9% were categorized as normal, 41.9% as osteopenic, and 12.2% as osteoporotic. However, when the TBS method was applied, the percentages changed to 33.8%, 41.9%, and 24.3%, respectively (Figure 4).

**Figure 5:**
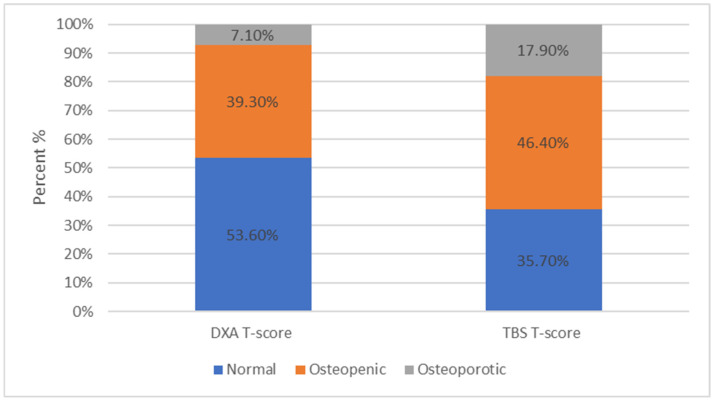
For patients with MGUS, 53.6% ranged within normal limits, 39.3% were characterized as osteopenic, and 7.1% as osteoporotic based on the DXA results. For patients with MGUS, 35.7% ranged within normal limits, 46.4% were characterized as osteopenic, and 17.9% as osteoporotic based on the TBS results.

For patients with MGUS, the distribution in the subcategories based on the DXA results was 53.6%, 39.3%, and 7.1%, for normal, osteopenic, and osteoporotic, respectively, whilst based on the TBS results, it was 35.7%, 46.4%, and 17.9%, respectively (Figure 5).

**Figure 6:**
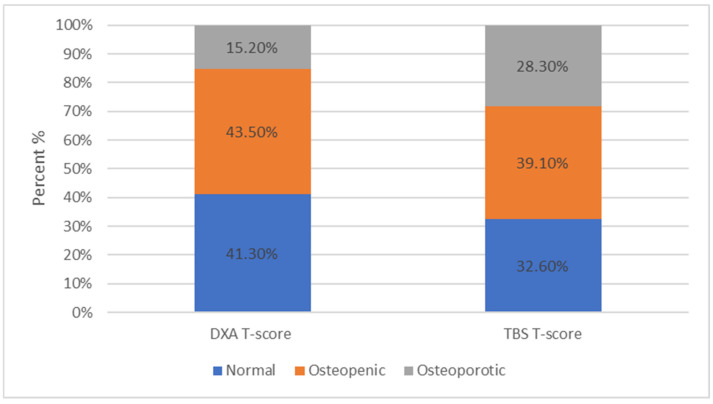
For patients with SMM, 41.3% ranged within normal limits, 43.5% were characterized as osteopenic, and 15.2% as osteoporotic based on the DXA scan. For patients with SMM, 32.6% ranged within normal limits, 39.1% were characterized as osteopenic, and 28.3% as osteoporotic based on the TBS results.

For patients with SMM, the distribution in the subcategories based on the DXA results were 41.3%, 43.5%, and 15.2% for normal, osteopenic, and osteoporotic, respectively, while it was were 32.6%, 39.1%, and 28.3%, respectively (Figure 6), based on the TBS results.

**Figure 7:**
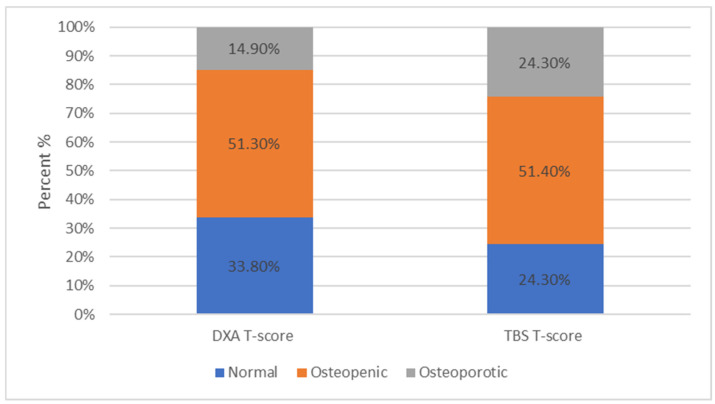
Among the controls, 25 (33.8%) were within normal limits, 38 (51.3%) were characterized as osteopenic, while only 11 (14.9%) were osteoporotic based on the DXA T-scores. Among the controls, 18 (24.3%) ranged within normal limits, 38 (51.4%), were characterized as osteopenic, and 18 (24.3%) as osteoporotic based on the TBS T-scores.

Finally, similar results were observed in the control group, as the percentages in each category were 33.8%, 51.3%, and 14.9%, for normal, osteopenic, and osteoporotic, respectively, based on the DXA T-scores, and 24.3%, 51.4%, and 24.3%, respectively, based on the TBS results (Figure 7).

It is evident from all the aforementioned analyses that the TBS T-score method provided higher percentages in the osteopenic and the osteoporotic categories compared to conventional DXA scans.

### 3.3. Correlations Between the Disease Subtype (SMM, MGUS) and Demographic Characteristics

The chi-square analysis revealed statistically significant relationships between the type of disease (SMM, MGUS) and the age group (<65, ≥65) (*p*-value = 0.018). For the age group ≥65, the proportion of patients with SMM was higher than the proportion of patients with MGUS. However, none of the other demographic characteristics (gender, group, BMI, smoking, dairy product consumption, family history of osteoporosis) were found to be associated with the type of disease (SMM, MGUS).

### 3.4. Multiple Correspondence Analysis (MCA)

**Figure 8:**
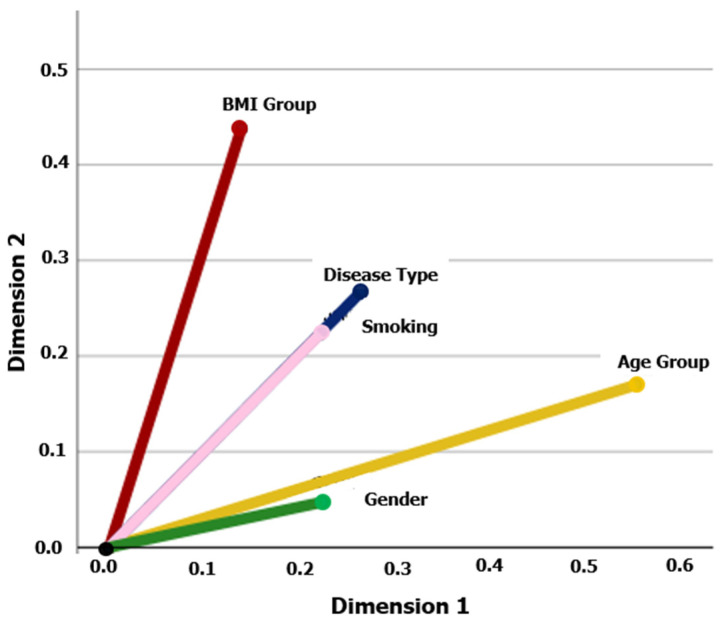
Multiple correspondence analysis of the patients’ characteristics. The analysis was performed for the following patients’ features: disease type (SMM, MGUS), gender, age group (<65, ≥65), smoking, and BMI group (<25, ≥25). The cosine of the angle between two vector lines represents the association between the corresponding variables. If two vector lines are close together, it suggests that the categories they represent are similar or positively associated. Conversely, if they are far apart, it indicates dissimilarity or a negative association. A 90° angle indicates that the variables are not related.

The application of MCA revealed interesting relationships between the type of disease (SMM, MGUS) and the demographic characteristics of the patients. Specifically, the type of disease showed a strong correlation with smoking, followed by BMI category, age group, and then gender (Figure 8).

## 4. Discussion

The selected measurement methods, DXA and TBS, are based on different biological mechanisms for evaluating bone health. DXA focuses on bone density, while TBS clarifies the trabecular microarchitecture. TBS captures information regarding the connectivity of trabecular struts. The loss of connectivity is a key factor in the mechanical stability of bones. Even if mineral content appears normal in a DXA scan, reduced trabecular connectivity, as estimated by TBS, can indicate a higher likelihood of fracture. This is particularly important in populations with age-related bone loss, where structural deterioration may occur without significant changes in BMD. TBS is sensitive to changes in bone quality due to the biological activity of osteoblasts and osteoclasts. DXA may not detect these changes, leading to differing assessments. Lastly, soft tissue composition can introduce variability in BMD results, affecting DXA measurements. TBS more directly reflects the underlying bone structure from the same DXA images, leading to discrepancies when soft tissue changes influence BMD interpretation but not the structural evaluation provided by TBS [29,30,31,32,33].

This study focuses on evaluating bone loss in patients with asymptomatic monoclonal gammopathy (MGUS and SMM) with the TBS score in addition to the conventional DXA scan. The study results provide valuable indications regarding the bone health of these patients and the possible implications for patient management.

This is consistent with previous studies that have shown that asymptomatic monoclonal gammopathy can be associated with an increased risk of osteoporosis and subsequent fractures [7,8,9,10,11,12,13,14,15,16,17,18].

Many studies have demonstrated that TBS can predict fragility fractures in cases of secondary osteoporosis caused by various conditions such as type I and II diabetes, hyperparathyroidism, Cushing’s syndrome, growth hormone deficiency, post-menopause hormone therapy, rheumatoid arthritis, spondyloarthritis, sarcopenia, chronic kidney disease, long-term glucocorticoid therapy, HIV infection, therapy with aromatase inhibitors, and neurofibromatosis [20,34].

Moreover, a low TBS score of the lumbar spine in newly diagnosed patients with symptomatic MM may be associated with an increased risk for fracture [35]. The continuous assessment of bone health in patients with MM is essential for making appropriate therapeutic decisions to improve quality of life. Interestingly, a study published in 2020 showed that patients with MGUS may be at a high risk for fractures even when DXA BMD and TBS indices are within normal values. This may be related to a thinness of the cortical bone rather than a loss of trabecular bone and underlines the need for supplemental tools in the assessment of bone health [36].

In our study, we observed a weak positive association between the T-Scores of measurements in the L1–L4 regions using DXA and TBS. This implies that the additional tool of TBS can lead to a different T-score category. More specifically, we observed that the classification of patients measured by DXA and categorized by T-score as normal, osteopenic, and osteoporotic changed when we implemented the TBS measurements. This resulted in patients initially evaluated as normal with DXA being reclassified as osteopenic or even osteoporotic with TBS. This observation was consistent in both the overall patient analysis, as well as within the two subgroups, i.e., MGUS and SMM. Therefore, the additional evaluation using TBS helps in better assessing skeletal health in these patients.

Additionally, the statistical analysis demonstrated a correlation between the age group of patients and the type of monoclonal gammopathy (SMM, MGUS). This suggests that age may affect the course of the disease and have potential impacts on bone health [37,38,39].

Furthermore, the MCA indicated that the type of monoclonal gammopathy has a strong correlation primarily with smoking and, secondarily, with BMI, age group, and finally, gender, aligning with other studies in the field [37,40,41].

In terms of study limitations, the number of participants was relatively small, as it was a pilot study and a robust power calculation was not conducted a priori. Additionally, our study did not conduct a follow-up evaluation after the initial assessment, thus we cannot evaluate long-term changes in the bone health of these patients. A more detailed assessment of bone health would also include imaging studies such as whole-body low-dose computed tomography and magnetic resonance imaging, along with an evaluation of biomarkers of bone metabolism [42]. Further studies are necessary to understand the exact mechanisms leading to bone loss in patients with asymptomatic monoclonal gammopathies and to develop more effective approaches for the prevention and management of osteoporosis.

Last but not least, an open question remains regarding the optimal management of patients with MGUS/SMM and osteopenia/osteoporosis. Should we implement the recommendations for osteopenia/osteoporosis management as in the general population or should we adopt a more intensified approach that resembles the bone-directed therapies administered in patients with symptomatic MM [43]? This consideration pertains to both the prompt administration of bisphosphonates or denosumab and the treatment schedule. The available data in the literature are scarce, whereas administration of three doses of zoledronic acid at 4 mg every 6 months improved BMD in patients with MGUS and concomitant osteopenia/osteoporosis in a small phase 2 study [44].

Furthermore, anti-myeloma regimens including combinations of proteasome inhibitors (bortezomib, ixazomib, carfilzomib) with either an anti-CD38 monoclonal antibody (daratumumab) or an immunomodulatory drug (lenalidomide, thalidomide) seem to improve indices of bone metabolism [45]. These data should be taken into consideration especially when discussing early treatment for a patient with smoldering myeloma at high risk for progression and bone loss by DXA BMD and/or TBS.

## 5. Conclusions

In conclusion, it was found that TBS values provide a supplemental understanding of the extent of bone loss due to underlying osteoporosis in patients with asymptomatic monoclonal gammopathy. Therefore, TBS represents a complementary assessment tool for bone health in this patient group that may aid in promptly initiating osteoporosis treatment and improving patient outcomes.

Clinicians should consider incorporating both TBS and DXA into routine bone health assessments for patients with MGUS and SMM to optimize risk stratification and management of skeletal fragility.

In addition, clinicians should be aware that patients with MGUS and SMM may have normal or mildly reduced BMD but still be at increased fracture risk due to compromised bone quality. A low TBS score in conjunction with normal BMD should prompt consideration of therapeutic interventions to reduce fracture risk.

## Figures and Tables

**Table 1:** Baseline characteristics of the participants in the study.

Characteristic	Patients (N = 74, 50.0%)	Controls (N = 74, 50.0%)
Gender		
Male	27 (36.5%)	27 (36.5%)
Female	47 (63.5%)	47 (63.5%)
Age—years (median, IQR)	65.5 (12)	62 (7)
Age Group		
<65	32 (43.2%)	48 (64.9%)
≥65	42 (56.8%)	26 (35.1%)
Height—cm (median, IQR)	161 (12)	161.5 (13)
Weight—kg (median, IQR)	74.5 (15)	72 (18)
BMI—kg/m^2^ (median, IQR)	28.8 (4)	27.3 (6)
BMI Group		
<25	14 (18.9%)	22 (29.7%)
≥25	60 (81.1%)	52 (70.3%)
Disease Type		
Smoldering Multiple Myeloma (SMM)	46 (62.2%)	0 (0.0%)
Monoclonal Gammopathy of undetermined significance (MGUS)	28 (37.8%)	0 (0.0%)
Osteoporosis History		
Family Positive History	8 (10.8%)	6 (8.1%)
Dairy Product Consumption		
No	47 (63.5%)	45 (60.8%)
Yes	27 (36.5%)	29 (39.2%)
Smoking		
No	57 (77.0%)	60 (81.1%)
Yes	17 (23.0%)	14 (18.9%)

## Data Availability

The original contributions presented in the study are included in the article further inquiries can be directed to the corresponding author.
